# Genomic and Micro-Evolutionary Features of *Mammalian 2 orthobornavirus* (Variegated Squirrel Bornavirus 1, VSBV-1)

**DOI:** 10.3390/microorganisms9061141

**Published:** 2021-05-25

**Authors:** Dániel Cadar, Jonas Schmidt-Chanasit, Dennis Tappe

**Affiliations:** 1 WHO Collaborating Centre for Arbovirus and Haemorrhagic Fever Reference and Research, Bernhard Nocht Institute for Tropical Medicine, 20359 Hamburg, Germany; jonassi@gmx.de (J.S.-C.); tappe@bnitm.de (D.T.); 2 Faculty of Mathematics, Informatics and Natural Sciences, Universität Hamburg, 20148 Hamburg, Germany

**Keywords:** bornavirus, zoonoses, animal husbandry, host switches, virus evolution, phylogeny

## Abstract

*Mammalian 2 orthobornavirus* (VSBV-1) is an emerging zoonotic pathogen discovered in several exotic squirrel species and associated with fatal human encephalitis. The dynamics of VSBV-1 spread and evolution in its presumed natural hosts are unknown. Here, we present the phylogeny, micro-evolution, cross-species transmission and spread of VSBV-1 at a temporal and spatial resolution within the limits of animal husbandry. The results showed that VSBV-1 can be classified into six distinct groups and that the most recent common ancestor of the known German strains emerged at least 20 years ago. We here demonstrate that the genetic diversity of the VSBV-1 groups is shaped primarily by in situ evolution and most of the amino acid changes are deleterious polymorphisms removed by purifying selection. Evidence of adaptive evolution has been found in the G and L genes which might have an influence on transmission fitness. Furthermore, there was also evidence for some form of adaptive changes in the glycoprotein which suggests that many sites might be subjected to positive pressure evolving under episodic directional selection, indicating past occurrence of positive selection. Host switching events were detected as dominant evolutionary mechanisms driving the virus-host associations. Virus spread by animal trade followed by subsequent local micro-evolution in zoos and holdings is responsible for diversifying strains. Time-resolved phylogeny indicated that Prevost’s squirrels might be the original squirrel species carrying and seeding the virus in Germany. This study provides the first insight into the ecology and micro-evolutionary dynamics of this novel viral pathogen in the captive exotic squirrel population under artificial ecological conditions (zoos and animal husbandry) and co-housing of different squirrel species.

## 1. Introduction

The variegated squirrel bornavirus 1 (VSBV-1; family Bornaviridae, species *Mammalian 2 orthobornavirus*) is an emerging zoonotic pathogen discovered in 2015 as the causative agent of fatal encephalitis of three men breeding exotic variegated squirrels in eastern Germany [1]. In 2018 and 2020, the virus was retrospectively shown to be responsible for two deadly encephalitis cases of animal caretakers in 2013 and 2007, respectively, in northern Germany [2,3]. The animal caretakers had occupational contact with exotic Prevost’s squirrels in the same zoological garden. While variegated squirrels (*Sciurus variegatoides*; family Sciuridae, subfamily Sciurinae) are native to Central America, Prevost’s squirrels (*Callosciurus prevostii*; family Sciuridae, subfamily Callosciurinae) are natural inhabitants of Southeast Asia. Epidemiological investigations in private holdings and zoological gardens in Germany and elsewhere in Europe detected VSBV-1 infection rates of 1.5% in captive Sciurinae and 8.5% in captive Callosciurinae squirrels, including one Asian Finlayson’s squirrel (*Callosciurus finlaysoni*; subfamily Callusciurinae) and one Asian striped squirrel (*Tamiops swinhoei*; subfamily Callosciurinae) [4,5]. Both variegated squirrels and Prevost’s squirrels were traded in complicated intermingling pathways between private breeders and zoos across Europe. Parts of this network in Germany have been revealed recently [6,7]. The origin of the virus is still unknown, but current time-resolved phylogeny and squirrel trade investigations suggested an introduction with Prevost’s squirrels to Germany between 1996–2003, followed by spread of the virus in different holdings and zoos with multiple spill-over infections to humans [7]. VSBV-1 has not been detected in wild European squirrels [8], and, so far, the virus has not been found in limited pilot investigations of Prevost’s squirrels currently conducted in Southeast Asia. This study provides a limited but unique glimpse into the ecology, micro-evolution, and perpetuation of this novel viral pathogen in captive squirrel populations under the artificial but exemplary condition of animal husbandry and co-housing of different squirrel species.

## 2. Materials and Methods

### 2.1. Genomic Characterization and Micro-Evolutionary Dynamics

All currently available 26 complete coding VSBV-1 sequences with known year of detection, host, and geographical origin available in databases were used in this study (Table S1). Sequences were aligned using the multiple alignments using fast Fourier transform (MAFFT) algorithm and the nucleotide and amino acid (AA) substitutions were visually inspected in Geneious Prime v2021.1.1 (Biomatters, Auckland, New Zealand). Phylogenetic trees were inferred using a Bayesian Markov chain Monte Carlo (MCMC) approach available in BEAST v1.8 [9] and a maximum likelihood (ML) method implemented in PhyML [10] with 1000 pseudo-replicates performed under the best fit nucleotide substitution model identified as the TN93+Γ using jModelTest 2 [11]. To assess whether there is sufficient temporal signal in the data to proceed with phylogenetic molecular clock analysis, regression of the root-to-tip divergence against the sampling time was inferred in TempEst [12]. Moreover, we estimated the rate of evolutionary rate (substitutions [subs] site^−1^ year^−1^), the time to most recent common ancestor (tMRCA) under a relaxed uncorrelated lognormal clock and a flexible demographic model (Gaussian Markov Random field Bayesian Skyride). An asymmetric model with Bayesian Stochastic Search Variable selection (BSSVS) was applied to identify statistically significant transition rates between host species. The Markov Chain Monte Carlo (MCMC) chain length was set to 50 million steps sampling after every 10,000 steps. The Bayesian maximum clade credibility (MCC) trees were visualized using FigTree v1.4.4 (http://tree.bio.ed.ac.uk/software/figtree/; accessed on 15 January 2021).

### 2.2. Selection Pressure Analyses on the VSBV-1 Genome

Codon-based models of evolution in an ML framework were used to evaluate the nature of the selection pressure acting on each VSBV-1 gene. It was estimated as a ratio of non-synonymous (*d_N_*) to synonymous (*d_S_*) nucleotide substitutions (*d_N_*/*d_S_* or ω ratio) per site for the detection of sites subjected to positive or negative selection or transient (episodic) selective pressures using four codon-based ML tools (SLAC, Single Likelihood Ancestor Counting; FEL, Fixed Effects Likelihood; MEME, Mixed Effects Model for Episodic Diversifying Selection Significance; FUBAR, Fast Unconstrained Bayesian AppRoximation) implemented in the Datamonkey web interface (http://www.datamonkey.org; accessed on 25 April 2021). We also employed aBSREL (Adaptive branch-site random effects likelihood) which uses an adaptive random effects branch-site model framework to test whether each branch has evolved under positive selection, using a procedure which infers an optimal number of rate categories per branch [13]. Predictions of potential glycosylation and furin cleavage sites were investigated on complete glycoprotein and polymerase sequences using NetNGlyc v1.0 and ProP v1.0 (http://www.cbs.dtu.dk/services/; accessed on 17 April 2021).

### 2.3. Genetic Diversity and Population Structure of VSBV-1

To search for evidence of host-specific adaptation, we identified AA that uniquely defined the major VSBV-1 groups. In order to visualize genealogical intra- and interspecies VSBV-1 relationships and the transmission history of VSBV-1 (i.e., origin from which holding or zoo), a haplotype network was constructed using the Minimum Spanning Network (MSN) method inferred by PopART package v1.7.2 [14].

### 2.4. Co-phylogeny Analysis of Virus-Host Co-evolution or Divergence

To study the history of a possible co-speciation of VSBV-1 and its hosts or the alternative hypothesis of host switches, we performed event-based co-phylogenetic reconciliation using CoRe-PA [15] and Jane 4 [16] software. The phylogenetic tree of hosts was obtained based on *cytochrome b* gene reconstruction using PhyML and the GTR+G model. The calculations using Jane 4 and CoRe-Pa assign event-based model with co-speciation, duplication, sorting and host switching events. Cost values must be set a priori in Jane 4, whereas CoRe-PA uses a parameter-adaptive approach to search for optimal cost values. Both methods compute minimal-cost reconstruction of the evolutionary history between hosts and viruses. For Jane 4 statistical significance was established by 500 random tip mapping permutations and 500 random parasite tree permutations with Yule beta parameter equal to −1. For Core-Pa the evaluation was done using 10,000 different event cost models. For significance evaluation, a randomization test with 1000 instances with randomized host-virus associations was performed.

## 3. Results

### 3.1. Genomic Characterization and Molecular Dynamics

**Figure 1:**
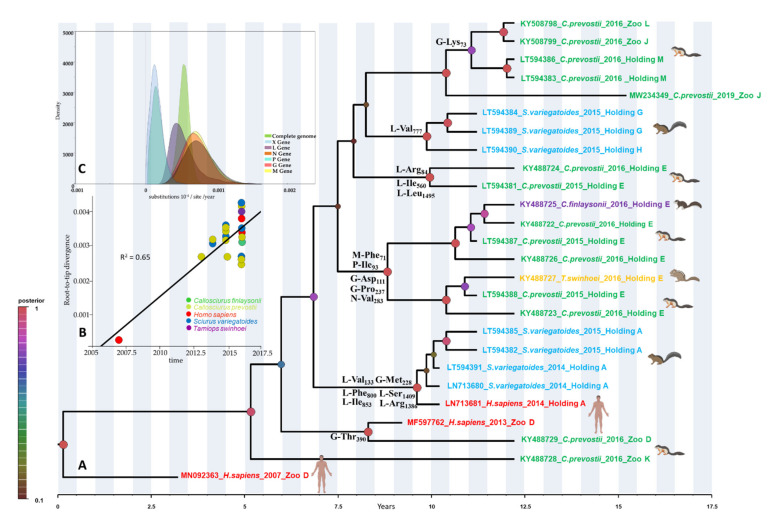
Phylogeny, sequence relatedness and micro-evolutionary rate of variegated squirrel bornavirus 1 (VSBV-1). (**A**) Bayesian maximum clade credibility (MCC) tree representing the timescale phylogeny reconstruction of VSBV-1. Bayesian posterior probabilities are indicated as colored circles at the nodes (see color codes). Time elapse is shown in the axis below the tree. Taxon information includes GenBank accession number, host, year of detection and origin. The fixed amino acid substitutions which determine the clustering of the groups are shown at the node of respective group. The first letter represents the protein in which the substitution occurred, followed by the amino acid change and its position in the respective protein. The silhouettes were created with BioRender.com. (**B**) Root-to-tip linear regression divergence plot of the VSBV-1 genome versus sampling year. (**C**) Estimates of micro-evolutionary rates (subs site^−1^ year^−1^) and 95% credible intervals for the complete genome and each gene of VSBV-1.

The genetic variation across the genome of VSBV-1 strains (Figure S1) was relatively heterogeneous but indicated that VSBV-1 has maintained a highly conserved genome since 2007. The identity matrices for the genome and individual genes were greater than 99.6% and 98.9% at the nucleotide and AA levels, respectively (Figure S2). Some structural and nonstructural genes (N, X, P, M) exhibited higher nucleotide than AA divergence. Interestingly, the greatest variation in both nucleotide and AA positions was observed in the glycoprotein (G) and polymerase (L) genes (Table 1 and Figure S1). Conversely, the M and P genes were the most conserved genes. The analysis of the AA substitutions on complete coding genomic sequences revealed both origin-specific (i.e., holdings or zoos) and host-specific point mutations across all VSBV-1 genes (Table 1 and Figure S1). Most of the host-specific non-synonymous substitutions have been observed in the G and L genes of Prevost’s squirrels and variegated squirrels, while for human cases such substitutions were found in the N, X, and P genes (Table 1). The similar topologies inferred by MCC from this study and ML phylogenies of the complete genome data revealed that all VSBV-1 strains fall into a monophyletic group, suggesting a single introduction event into Germany (Figure 1A) as previously reported [7]. Although the VSBV-1 diversity appears to have emerged in the last decade (tMRCA of each individual group showed a timeframe between August 2008–July 2011; 95% high-probability density [95% HPD] February 2007–July 2012), the phylogeny suggests relative longer circulation of VSBV-1 in Germany (Figure 1A). Based on the available data, we estimated that the virus likely spread in Germany from an ancestor that existed around the year 2000 (95% HPD for 1993 to 2004; posterior probability [pp] = 0.99).

However, the limited number of available sequences and the lack of VSBV-1 data back to the first known human case in 2007 reduces our ability to predict previous events and virus variant circulation between holding and zoos. The detailed analysis of the German strains demonstrated that VSBV-1 forms six major groups (Figure 1A) defined by place of sampling (i.e., holdings or zoos). These results further provide strong support for in situ evolution of the virus. Furthermore, the clustering of the human-derived VSBV-1 strains with the respective squirrel-derived virus strains from the same local settings (holding A and zoo D), suggests individual spill-over infections from squirrels to humans (Figure 1A), as previously reported [7]. The most likely host species of the common ancestor of all VSBV-1 strains was estimated to be the Prevost’s squirrel (pp = 0.92) (Figure 1A), as seen recently [7] and corroborated here. Our host-based transition phylogeny revealed that VSBV-1 groups have possibly arisen from a Prevost’s squirrel-derived VSBV-1 strain, further supporting potential introduction into Germany by this squirrel species. 

In total, 17 fixed AA substitutions were revealed that supported phylogenetic grouping of the German VSBV-1 strains. Out of these, one AA substitution was observed in the N, P and M gene each, while five were found in the G gene, and nine were seen in the L gene, respectively (Figure 1A). The genome-based data set exhibited a strong temporal signal (R2 = 0.65, *P* < 0.001) and the coefficient of rate variation supported the use of a relaxed clock model. By plotting root-to-tip divergence as a function of sampling time, an accumulation of nucleotide substitutions was observed over the sampling time (Figure 1B). The evolutionary time-resolved analyses for each individual gene exhibited a mean rate in a narrow range of 2.41 × 10^−4^ to 7.33 × 10^−4^ subs site^−1^ year^−1^ (Figure 1C). The N gene exhibited the highest and the X gene the lowest evolutionary rate, while the complete genome exhibited a rate of 2.86 × 10^−4^ subs site^−1^ year^−1^ (Figure 1C). These rates are comparable to that previously estimated for other bornaviruses or members of order *Mononegavirales* [17,18,19].

### 3.2. Selection Pressure Analyses on the VSBV-1 Genome

The nature of the selection pressure acting on the VSBV-1 genes shows that the overall ratio of non-synonymous to synonymous substitutions were between 0.007 and 0.031, indicating that most AA changes are deleterious polymorphisms removed by purifying selection (Table 2). Significant evidence of adaptive changes was found only at the AA site 238 of the G gene and the AA site 1191 of the L gene (Table 2). A positively selected AA (Ser_238_Leu) detected in the glycoprotein of VSBV-1 was present in two Prevost’s squirrels (KY488723 and KY488724) and one Swinhoe’s striped squirrel (KY488727), while another positively selected AA (Lys_1191_Leu/Arg) detected in the polymerase was present in two Prevost’s squirrels (LT594381 and KY488724), all originating from the same holding E (Figure 1A). The Ser_238_Leu is a conservative change which caused a change from a polar, uncharged, to a nonpolar, hydrophobic uncharged AA.

The conservative AA change Lys_1191_Leu/Arg was located in the large downstream ORF towards the C terminus of the polymerase and caused a change from a positively charged, to a nonpolar, hydrophobic uncharged and to a positively charged AA, respectively. Both AA changes in the G and L protein were not located at a potential furin cleavage site or at N-glycosylation sites. Furthermore, there was also evidence for some form of adaptive changes (episodic positive/diversifying selection) in the glycoprotein which suggests that many sites in the VSBV-1 genes (Thr_170_Ala–variegated squirrel; Ser_170_Asn–Prevost’s/Finlayson’s squirrel/human; Asn_188_Asp/Ser–Prevost’s squirrel; Asn_390_Thr–Prevost’s squirrel/human; Asn_390_Ser–variegated squirrel) identified by FUBAR and MEME may be subjected to positive pressure evolving under episodic directional selection (Table 2). Using the aBSREL method, we were able to detect a group-specific (KY488724/LT594381) micro-evolutionary pattern in our dataset (*p* = 0.0002; ω_1_ = 0.0993).

### 3.3. Analysis of Possible Virus-Host Co-Evolution or Host Switching Events

**Figure 2:**
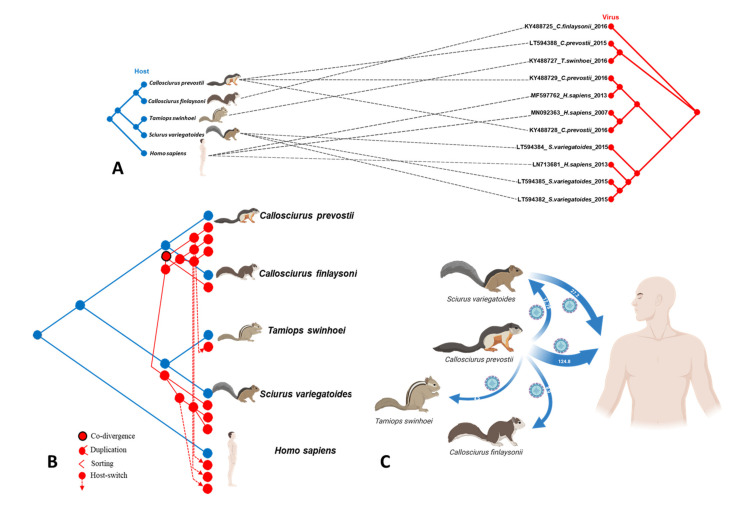
Host-parasite systems of mammalian hosts and VSBV-1 and transmission strengths of squirrel-derived VSBV-1 strains. (**A**) Co-phylogenetic reconstruction of the relationship between VSBV-1 and its known hosts based on complete coding-region alignments of VSBV-1 and taxonomic relationships of hosts based on the mtDNA *cyt b* gene phylogeny using CoRe-PA software. The blue tree represents the phylogeny of the hosts, while the red tree the phylogeny of VSBV-1. (**B**) Representation of the most parsimonious co-evolutionary reconciliation scenario for VSBV-1 and its known hosts proposed by CoRe-PA. Four evolutionary events are denoted: co-divergence, duplication, sorting, and host switches. Dashed red lines indicate distant host jump events. (**C**) Connectivity and magnitude of transmission network of VSBV-1 in host species. Arrows indicate the origin and direction of transmission of VSBV-1 between host species and arrow widths are proportional to the strength of the transmission rate (Bayes factor, BF). Figure 3C and the silhouettes of figures were created with BioRender.com.

Using CoRe-PA and Jane 4, we tested the hypothesis of VSBV-1/host co-speciation by using reconciliation analysis, presented along with their tanglegram depicting host-virus associations (Figure 2A,B). Event-based co-phylogeny methods apply cost schemes to different evolutionary events to test the level of congruence between the host and virus trees. By evaluating 5000 random cost schemes, CoRe-PA computed the most parsimonious reconstruction and predicted the frequencies for co-evolutionary events, including co-speciation, host switching, duplication, and sorting. CoRe-Pa yielded 23 reconstructions, with two co-divergence events, four duplications, zero sortings, and four host switchings. The second-best solution had a similar number of events (Table 3). Results from Jane 4 using the default cost scheme revealed a total of 14 costs, indicating two co-divergence events, three duplications, one sorting, and five host switchings (Table 3 and Supplementary Materials Figure S3). Thus, both CoRe-Pa and Jane 4 indicated that co-speciation likely did not play a role in the observed associations between VSBV-1 and its hosts, and that host switchings and subsequent adaptations have occurred in the limited time-span of the virus micro-evolution investigated here instead.

### 3.4. Transmission Events of VSBV-1 to Different Hosts

**Figure 3:**
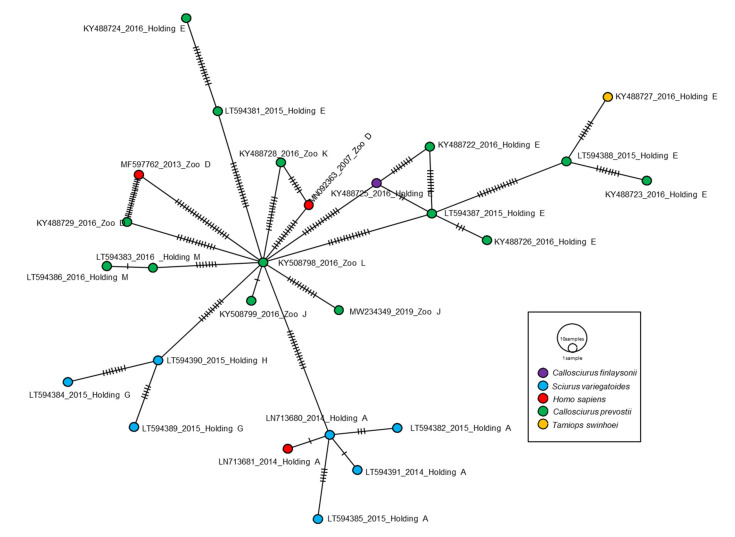
A minimum-spanning network constructed from a coding-complete genome alignment of different VSBV-1 strains. Each colored vertex represents a sampled viral haplotype, with different colors indicating the different hosts. The size of each vertex is relative to the number of sampled viral strains and the dashes on branches show the number of mutations between nodes.

The VSBV-1 phylogeny is notable for a clustering in which the dominant groups connect viruses sampled from multiple time points from the same source (Figure 1A). We used a BSSVS procedure to identify viral host switches (cross-species transmission over evolutionary time) between humans and squirrel species that took place along the branches of the MCC tree and calculated the Bayes factor (BF) to estimate the significance of these transitions events (i.e., non-zero rates supported by a BF of > 3). Our ancestral reconstruction revealed highly supported host switches of VSBV-1 between the four squirrel species and humans (Figure 2C). VSBV-1 from Prevost’s squirrels may have been transmitted to the other three squirrel species and humans with a high BF between 4 and 124. There was a notable host jump from Prevost’s squirrels to humans, and from Prevost’s squirrels to variegated squirrels, with mean transmission BFs of 124 and 27, respectively (Figure 2C). These results indicate that Prevost’s squirrel-derived VSBV-1 were transmitted more frequently than VSBV-1 strains from other squirrel species. A minimum-spanning haplotype inferences further strengthen that the VSBV-1 strains seem to belong to multiple distinct groups, all of them having likely emerged from Prevost’s squirrel-derived strains (Figure 3).

## 4. Discussion

The human impact on sylvatic or semi-sylvatic ecosystems as well as the trade of wild animal species facilitates contact of (novel) pathogens with susceptible (new) hosts. Understanding the ecology and genetic driving forces that contribute to the emergence and spread of zoonotic pathogens is important for the implementation of preventive strategies. In this study, we aimed to investigate the genomic and micro-evolutionary processes of VSBV-1 that were seen during infection of multiple exotic squirrel species and humans in the artificial but exemplary situation of animal husbandry and co-housing of squirrel species which live under natural conditions on different continents. Moreover, we aimed to shed more light on the possible origin of VSBV-1 infecting exotic squirrels in German holdings by novel phylogeny constructions, co-speciation analyses, transmission rate strength calculations, and haplotype network inferences.

The phylogeny constructed here showed a spatial differentiation of VSBV-1 strains that resulted in the phylogenetic clustering in the form of six distinct groups defined by the geographic place of sampling (i.e., holdings or zoos). The existence of such groups is likely the result of adaptation to the local artificial ecological conditions. Hence, the adaptation of VSBV-1 to naïve host populations in captivity can lead to the emergence of local virus strains or variants, and the most likely scenario for VSBV-1 might be enzootic maintenance (in situ evolution). This assumption is further supported by the presence of some fixed AA residues involved in the formation of these six distinct groups defined by their geographic origin.

The overall low ratio of non-synonymous to synonymous substitutions indicates that most AA changes are deleterious polymorphisms removed by purifying selection, likely as results of genetic drift. We found strong evidence of adaptive evolution only at codon 238 of the G gene and at codon 1191 of the L gene. Although the function of these residues remains to be determined, mutations in the G protein and/or L protein (polymerase) have exhibited enhanced neurovirulence in the related Borna Disease virus 1 (BoDV-1) in adult and newborn Lewis rats [20]. In a recent study, following isolation from primary cell cultures and passage in Lewis rats, VSBV-1 was shown to be genetically highly stable [21]. However, within three VSBV-1 isolates examined, at least one AA exchange occurred in the viral glycoprotein. These exchanges mostly reverted after passage in Lewis rats at least for two isolates and were interpreted as a necessary adaption process to allow efficient replication in cell cultures of another species’ origin [20]. Furthermore, in our study there was also evidence for some form of adaptive changes in the glycoprotein which suggests that many sites might be subjected to positive pressure evolving under episodic directional selection, indicating past occurrence of positive selection. Adaptive evolution frequently occurs in episodic bursts, localized to a few sites in a gene, and to a limited number of lineages in the phylogenetic tree [22]. Using branch-site specific analysis we observed that the diversification pressure was strong in one of the VSBV-1 groups (KY488724/LT594381).

VSBV-1 infected captive exotic squirrel species of the Sciurinae and Callosciurinae subfamilies were shown to be asymptomatic, thus displaying characteristics of natural reservoir hosts [4,5]. The finding that the VSBV-1 infection rates in Southeast Asian Callosciurinae squirrel subfamily members in European holdings are considerably higher than the infection rates in exotic squirrels from the Sciurinae subfamily [4,5] suggested that Prevost’s squirrels might be the original squirrel species carrying the virus in Germany and the natural reservoir for VSBV-1, and thus Southeast Asia a possible geographical region of the virus’ origin. However, certain limitations should be considered because the diversity of VSBV-1 detected is biased by limited sampling, and virus variants from the introduction event into Germany until the first known human infection in 2007 have certainly been missed. Therefore, due to the limited availability of full VSBV-1 genomic sequences from different hosts, also the host species analysis should be interpreted with caution. Recently, time-resolved phylogeny indicated that Prevost’s squirrels were putatively responsible for the introduction of VSBV-1 to Germany [7], a finding which we here underscore by further phylogenetic investigations, host transition calculation results with strong BFs, and respective haplotype network analyses. Haplotype networks do not necessarily represent evolutionary history reconstructions unless other corroborating evidence is available [14], such as our previous [7] and the current phylogeny investigations. Our estimates suggested that VSBV-1 emerged in Germany at least 20 years ago. Our current VSBV-1 group-specific tMRCA analysis confirms our previous common VSBV-1 tMRCA calculation [7]. It is important to note, however, that the accuracy of the tMRCA calculation for VSBV-1 could be influenced by the limited amount of complete sequence data, as well as the short and unbalanced time span that can lead to underestimation of the lengths of long phylogenetic branches.

By co-phylogenetic reconciliation analysis, host switching was detected as the dominant mechanism driving the virus-host associations during animal trade and co-housing of different species in captivity. No hints for a co-speciation were found. When analyzed by host switching, both squirrel inter-genus and intra-genus transmissions have occurred. This suggests that VSBV-1 may switch its hosts (by spill-over infections) if the environmental conditionals are favorable, such as during co-housing or by contact to human caretakers, apparently without preferential transmission between closely or more distantly related host species. Our results do not exclude a possible co-speciation of VSBV-1 and different exotic squirrels or other host species in the wild. Field studies for the presence of VSBV-1 in wild exotic squirrel populations are therefore still essential to resolve the question of the virus’ natural host animal range and its geographic origin. This is underlined by recent investigations which showed that the animal trade to Germany was mainly fueled by a squirrel farm in Central America where Prevost’s squirrels were kept beside variegated squirrels [7]. Thus, variegated squirrels or a still unknown Central American animal infected with VSBV-1 might be the natural reservoir hosts nonetheless, leading to a spill-over infection of Prevost’s squirrels destined for export to Germany at the squirrel farm. How individual squirrels become infected during trade and husbandry and under which conditions host switches of the virus during co-habitation of different species occur in captivity also remains to be elucidated. Sciurinae and Callosciurinae squirrels develop high viral loads in the central nervous system and organs capable of secretion and excretion. Many animals stayed non-infected despite close contact with infected squirrels [4,5]. It is assumed that humans might become infected by scratches and bites of the animals [1] and/or after mucosal contact [6].

Our analysis only represents a snapshot into the last evolutionary events identified and as such does not represent the complete evolutionary history of VSBV-1. It should also be noted that such events are dependent on the relationships observed in the reconstructed phylogeny trees and could appear differently once more VSBV-1 strains are found and compared with each other.

## 5. Conclusions

Taking advantage of the artificial circumstances of animal husbandry, we were able to gain insights into adaptive and other micro-evolutionary processes in different exotic squirrel hosts that form the basis for future studies on the ecology and evolution of this zoonotic emerging viral pathogen under natural conditions. The limited number of available VSBV-1 sequences and the lack of virus data from other European countries and other continents reduce our ability to infer the spatio-temporal pattern of the virus’ spread and emergence, and thus the evolutionary history of VSBV-1. 

## Figures and Tables

**Table 1:** Host-specific non-synonymous substitutions among VSBV-1 genomes.

Host	VSBV-1 Protein					
	N	X	P	M	G	L
*Homo sapiens*	Leu96Cys, Glu160Asp, Glu275Gly	Gly30Arg	Pro35Ser, Ser51Phe, Val122Ile	Asp43Asn	Asp43Asn, Glu185Ala	Gly54Glu, Arg88His, Gly1084Arg
*Callosciurus prevostii*	Val147Ile, Thr254Ile		Val79Ile	Ser19Leu, Glu61Lys, Ala106Val	Ser19Leu, Glu61Lys, Ala106Val, Val313Ile, Asn188Asp, Gly196Asp	Lys84Arg, Lys141Glu, Val560Ile, Ile1074Val, Lys1191Arg/Leu, Gln1307His, Ser1495Leu
*Sciurus* *variegatoides*		Pro21Ser, Thr37Ile		Ser165Gly	Ser165Gly, Thr170Ala, Asn390Ser, Arg244Lys, Asn188Ser	Ala165Thr, Glu164Lys, Cys287Tyr

**Table 2:** Selection pressure analysis showing the position of positively selected codons in the alignment of VSBV-1 genomes. Codons corresponding to amino acid positions found to be positive by at least three approaches are listed in the table.

Gene	SLAC *	FEL *	MEME *	FUBAR ^#^	Amino Acid Substitution	dN/dS
**N**						0.007
**X**						0.019
**P**						0.013
**G**		**238**	**238**	170, 175, 188, **238**, 390	Thr170Ala, Ser175Asn/Ile, Asn188Asp/Ser, **Ser238Leu**, Asn390Thr/Ser	0.031
**M**						0.009
**L**		**1191**	**1191**		**Lys1191Leu/Arg**	0.014

*p-values less than 0.05 and **^#^** posterior probabilities (PP) > 0.8 were considered statistically significant; SLAC: Single Likelihood Ancestor Counting; FEL: Fixed Effects Likelihood; MEME: Mixed Effects Model for Episodic Diversifying Selection Significance; FUBAR: Fast Unconstrained Bayesian AppRoximation; *d_N_/d_S_*: ratio of non-synonymous substitutions per non-synonymous site to synonymous substitutions per synonymous site.

**Table 3:** Results of reconciliation analyses for VSBV-1 and its hosts using CoRe-Pa and Jane 4.

**Core-Pa**	**Reconstruction (q_c_)**	**Total Cost**	**Co-Speciation** **(Cost)**	**Duplication** **(Cost)**	**Host-Switch** **(Cost)**	**Sorting** **(Cost)**	***p* Value** **(Tip Mapping)**	***p* Value** **(Parasite Tree)**
	1 (0.023)	23	1 (0.106)	4 (0.169)	5 (0.552)	0	<0.015	<0.015
	2 (0.037)	23	2 (0.189)	2 (0.195)	3 (0.557)	1 (0.08)	<0.015	<0.015
**Jane 4**	**Cost Set**	**Total Cost**	**Co-Speciation**	**Duplication**	**Host-Switch**	**Losses**	**Failure to Diverge**	***p* Value**
	1	14	2	3	5	1	0	0.08
	2	14	2	3	5	1	0	0.08

## Data Availability

Not applicable.

## Supplementary Materials

The following are available online at https://www.mdpi.com/article/10.3390/microorganisms9061141/s1, Figure S1: Amino acid substitutions of VSBV-1 proteins from different holdings and zoos; Figure S2: Nucleotide (a) and amino acid (b) sequence identity of VSBV-1; Figure S3: Co-phylogenetic analysis of the VSBV-1/hosts mutualism conducted with Jane 4 using phylogenies including representative number of representative parasites and their known host species; Table S1: Collection date, host and origin of VSBV-1 genomes used in the study.
