# Supplementary material for: Genomic and Micro-Evolutionary Features of Mammalian 2 orthobornavirus (Variegated Squirrel Bornavirus 1, VSBV-1)
Source: Microorganisms. 2021 May 25;9(6):1141. doi: 10.3390/microorganisms9061141 (PMC8227138; doi:10.3390/microorganisms9061141)
Supplement: Supplementary file 1 [file microorganisms-09-01141-s001.zip › microorganisms-1239024-supplementary/microorganisms-1239024 suppl resub/Figure S3.pptx]

## Slide 1
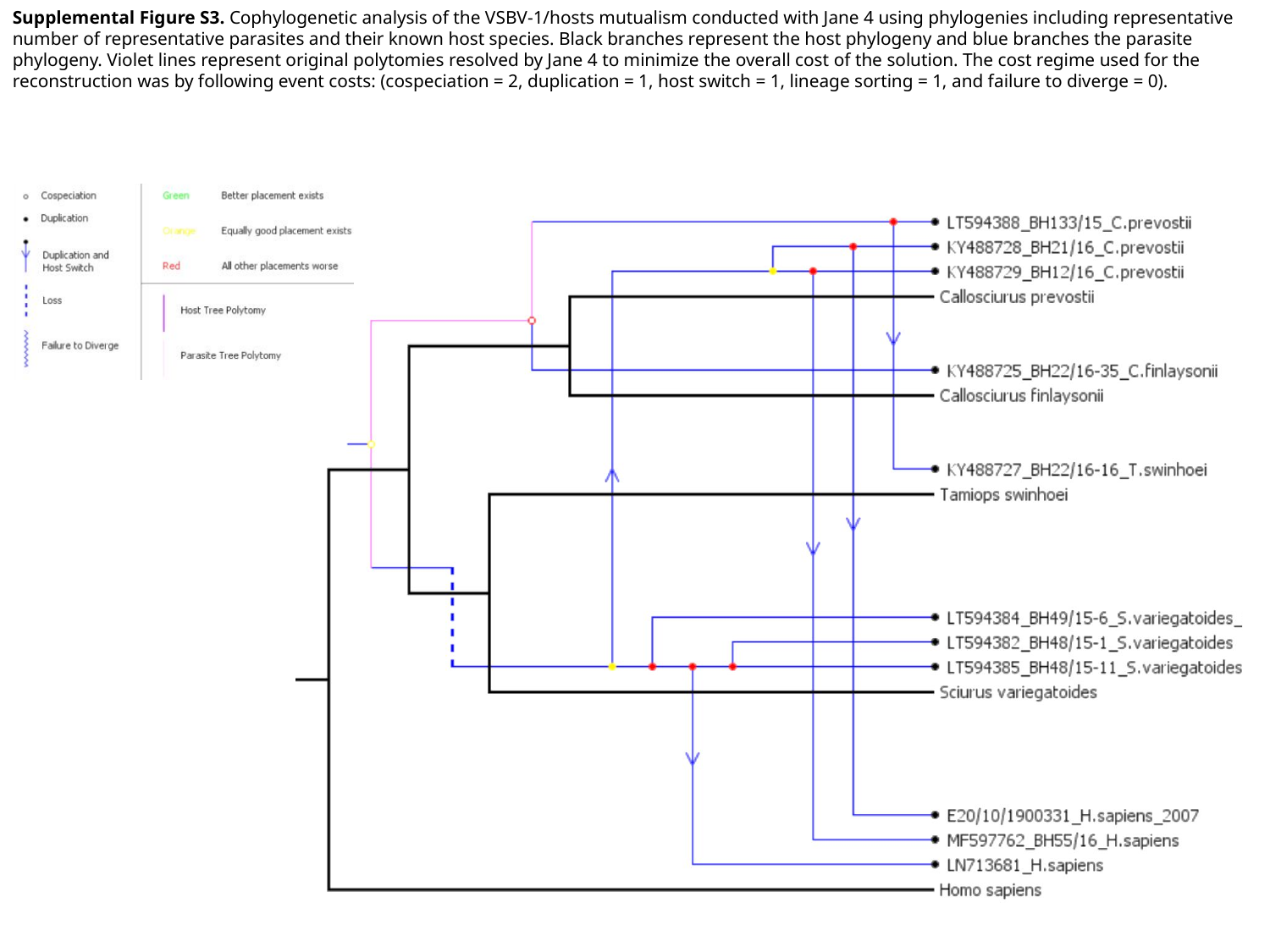

Supplemental Figure S3. Cophylogenetic analysis of the VSBV-1/hosts mutualism conducted with Jane 4 using phylogenies including representative number of representative parasites and their known host species. Black branches represent the host phylogeny and blue branches the parasite phylogeny. Violet lines represent original polytomies resolved by Jane 4 to minimize the overall cost of the solution. The cost regime used for the reconstruction was by following event costs: (cospeciation = 2, duplication = 1, host switch = 1, lineage sorting = 1, and failure to diverge = 0).
