# Supplementary material for: Genomic and Micro-Evolutionary Features of Mammalian 2 orthobornavirus (Variegated Squirrel Bornavirus 1, VSBV-1)
Source: Microorganisms. 2021 May 25;9(6):1141. doi: 10.3390/microorganisms9061141 (PMC8227138; doi:10.3390/microorganisms9061141)
Supplement: Supplementary file 1 [file microorganisms-09-01141-s001.zip › microorganisms-1239024-supplementary/microorganisms-1239024 suppl resub/Table S1.pdf]

Supplemental Table S1. Collection date, host and origin of VSBV-1 genomes used in the study.

GenBank acc. no.	Year of detection	Host	Location	Region
KY488723	2016	*C. prevostii*	Holding E	Northwest Germany
KY488727	2016	*T. swinhoei*	Holding E	Northwest Germany
LT594388	2015	*C. prevostii*	Holding E	Northwest Germany
KY488724	2016	*C. prevostii*	Holding E	Northwest Germany
LT594381	2015	*C. prevostii*	Holding E	Northwest Germany
KY488726	2016	*C. prevostii*	Holding E	Northwest Germany
KY488722	2016	*C. prevostii*	Holding E	Northwest Germany
LT594387	2015	*C. prevostii*	Holding E	Northwest Germany
KY488725	2016	*C. finlaysonii*	Holding E	Northwest Germany
LT594384	2015	*S. variegatoides*	Holding G	East Germany
LT594389	2015	*S. variegatoides*	Holding G	East Germany
LT594390	2015	*S. variegatoides*	Holding H	North Germany
LT594385	2015	*S. variegatoides*	Holding A	East Germany
LT594382	2015	*S. variegatoides*	Holding A	East Germany
LN713680	2014	*S. variegatoides*	Holding A	East Germany
LT594391	2014	*S. variegatoides*	Holding A	East Germany
LN713681	2013	*H. sapiens*	Holding A	East Germany
KY488728	2016	*C. prevostii*	Zoo K	East Germany
KY488729	2016	*C. prevostii*	Zoo D	North Germany
MF597762	2013	*H. sapiens*	Zoo D	North Germany
MN092363	2007	*H. sapiens*	Zoo D	North Germany
KY508799	2016	*C. prevostii*	Zoo J	Southwest Germany
MW234349	2019	*C. prevostii*	Zoo J	Southwest Germany
LT594383	2016	*C. prevostii*	Holding M	The Netherlands
LT594386	2016	*C. prevostii*	Holding M	The Netherlands
KY508798	2016	*C. prevostii*	Zoo L	Southwest Germany
