# Supplementary figures and images for: Genomic and Micro-Evolutionary Features of Mammalian 2 orthobornavirus (Variegated Squirrel Bornavirus 1, VSBV-1)
Source: Microorganisms. 2021 May 25;9(6):1141. doi: 10.3390/microorganisms9061141 (PMC8227138; doi:10.3390/microorganisms9061141)

## Slide 1
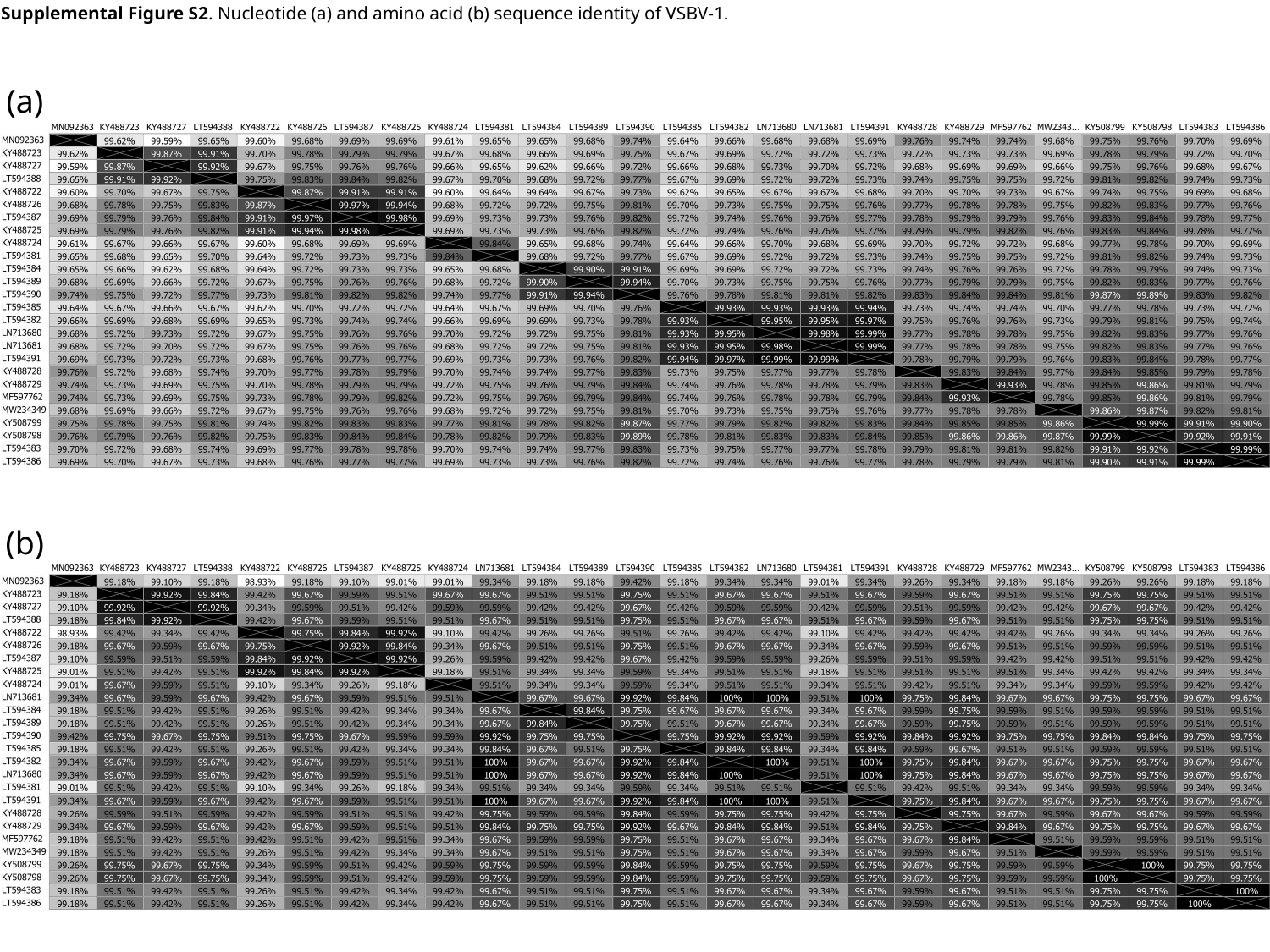

Supplemental Figure S2. Nucleotide (a) and amino acid (b) sequence identity of VSBV-1.
(a)
(b)

Supplement: Supplementary file 1 [file microorganisms-09-01141-s001.zip › microorganisms-1239024-supplementary/microorganisms-1239024 suppl resub/Figure S2.pptx]
